# Phase-Sensitive Measurements of Depth-Dependent Signal Transduction in the Inner Plexiform Layer

**DOI:** 10.3389/fmed.2022.885187

**Published:** 2022-06-01

**Authors:** Clara Pfäffle, Hendrik Spahr, Katharina Gercke, Léo Puyo, Svea Höhl, David Melenberg, Yoko Miura, Gereon Hüttmann, Dierck Hillmann

**Affiliations:** ^1^Institute of Biomedical Optic, University of Lübeck, Lübeck, Germany; ^2^Medical Laser Center Lübeck GmbH, Lübeck, Germany; ^3^Department of Ophthalmology, University of Lübeck, Lübeck, Germany; ^4^Airway Research Center North, Member of the German Center for Lung Research, Grosshansdorf, Germany; ^5^Center of Brain, Behavior and Metabolism (CBBM), University of Lübeck, Lübeck, Germany; ^6^Thorlabs GmbH, Lübeck, Germany; ^7^Department of Physics, Vrije Universiteit Amsterdam, Amsterdam, Netherlands

**Keywords:** optoretinography, optical coherence tomography, phase-sensitive OCT, functional imaging, inner plexiform layer, retina

## Abstract

Non-invasive spatially resolved functional imaging in the human retina has recently attracted considerable attention. Particularly functional imaging of bipolar and ganglion cells could aid in studying neuronal activity in humans, including an investigation of processes of the central nervous system. Recently, we imaged the activity of the inner neuronal layers by measuring nanometer-size changes of the cells within the inner plexiform layer (IPL) using phase-sensitive optical coherence tomography (OCT). In the IPL, there are connections between the neuronal cells that are dedicated to the processing of different aspects of the visual information, such as edges in the image or temporal changes. Still, so far, it was not possible to assign functional changes to single cells or cell classes in living humans, which is essential for studying the vision process. One characteristic of signal processing in the IPL is that different aspects of the visual impression are only processed in specific sub-layers (strata). Here, we present an investigation of these functional signals for three different sub-layers in the IPL with the aim to separate different properties of the visual signal processing. Whereas the inner depth-layer, closest to the ganglion cells, exhibits an increase in the optical path length, the outer depth-layer, closest to the bipolar cell layer, exhibits a decrease in the optical path length. Additionally, we found that the central depth is sensitive to temporal changes, showing a maximum response at a stimulation frequency of around 12.5 Hz. The results demonstrate that the signals from different cell types can be distinguished by phase-sensitive OCT.

## 1. Introduction

Non-invasive, cellular imaging of the neuronal activity in humans is compelling for clinical diagnosis and basic science (1). Currently, only optical methods may achieve the necessary resolution for this purpose. In addition, the retina is the only site, where the central nervous system is directly accessible for optical imaging. Besides being part of the central nervous system (CNS) and thereby providing diagnostic information on neuronal diseases, the initial information processing of visual impressions, which is mainly performed by the bipolar, amacrine, and ganglion cells, can be studied here (2–4). Each of these cell classes can be further divided into different functional cell types, which are sensitive to specific features of the visual signal. Currently, around 13 different types of bipolar and around 30 types of ganglion cells are known. They are responsible for processing different aspects for the visual information, e.g., brightness, color, as well as spatial and temporal variations, thus splitting the visual information into different feature channels (2, 5).

In recent years, there has been extensive research to identify these different cell types and assign them to different functional tasks (2–5). Although many types have already been classified, there are still gaps in the knowledge about these different cells. To access these cell classes one can use retinal imaging with functional contrast. Suitable functional signals in the inner layers of the retina could be detected with different imaging methods [reviewed in (6)]. Techniques capable of identifying different cell types make, for example, use of calcium imaging (7, 8). However, these methods require insertion of genetically encoded calcium indicators and are thus only available for animal studies.

Several other, non-invasive techniques could be and partially were applied to the living human eye. For example, electroretinogram (ERG) measurements provide information about the overall activity of the retina (9), but cannot spatially resolve contributions from different cell types in the retina. Furthermore, neurovascular coupling using laser Doppler flowmetry (10, 11) or optical coherence tomography angiography (OCTA) (12) can measure increased blood flow when the retina is stimulated. This has already been used to measure the sensitivity of the retina to temporal changes of the visual signal in cats (10, 11) and mice (12). The techniques may also achieve a relatively high resolution by measuring blood flow in the capillaries. Still, these techniques do not allow distinguishing of the functional contributions of individual functional cell types.

Furthermore, it has been shown that light-stimulated activation of the retinal function may lead to a change in reflectivity of the IPL that can be measured with optical coherence tomography (OCT). These changes in reflectivity due to functional activation could be demonstrated in different animals, for example, *ex vivo* in frog retina preparations (13), in paralyzed tree shrews (14), and in a few studies even *in vivo* in human (15). However, such measurements of changing reflectivity exhibit a high noise level, making it particularly challenging to extract the functional information from *in-vivo* measurements.

One technique, that emerged in the past years for non-invasive detection of the functional changes is phase-sensitive OCT. This technique can detect variations in the optical path length between the different retinal layers with a resolution of a few nanometers (16). Additionally, due to the tomographic character of OCT, such variations can be assigned axially and laterally to specific locations with a resolution better than 10 μm, which makes it in principle possible to assign functional changes to single cells. This cellular phase-based functional contrast has so far been demonstrated for the photoreceptor outer segments (OS) with several OCT techniques including Fourier-domain scanning (17–20), line-field (21), and full-field (16, 22) systems. Recently, by using a full-field swept-source OCT (FF-SS-OCT) system, we were able to show functional phase-changes in the inner plexiform layer (IPL) (23), where the synaptic connections of the bipolar, amacrine, and ganglion cells are located. However, low contrast, an order of magnitude smaller optical path length changes, and heart-beat induced pulsatile retinal motion make this endeavor extremely challenging in the inner neuronal layers. In the first demonstration of functional signals in the IPL, the resulting noise was minimized by extensive averaging of the phase over several voxels in the axial dimension. Due to this averaging, the contributions from all different functional bipolar/ganglion cells were integrated and no differentiation of the functional change in the IPL was possible. Unfortunately, the structural sensitivity of FF-SS-OCT has been insufficient to image individual ganglion or bipolar cells, although single ganglion cell imaging was demonstrated for other OCT systems by averaging hundreds of images (24).

Here, we use a recently demonstrated phase evaluation that takes advantage of the statistical properties of random and systematic phase changes to minimize phase noise (25) and use the stratification of the synaptic connections to differentiate signals from different cell types in the living human retina. With this phase evaluation, phase averaging in the axial dimension can be avoided to a large extent, so that the functional changes could be assigned to their origin in the retina with much higher accuracy allowing the evaluation of sub-layers. At the same time, the reduced noise levels enable a better investigation of the lateral dependency of the signal in the IPL. Although, single cell functional imaging of the inner neurons is still not possible due to the limited structural sensitivity, we could use the increased axial resolution and the improved signal-to-noise ratio (SNR) to distinguish the contributions of different functional cell types to the change in optical path length. To this end, we exploit the interconnection behavior of the different cell types in the IPL. Since certain functional bipolar and ganglion cell types form their synaptic connections only in a specific depth of the IPL, the corresponding properties of the visual impression are also processed at a specific depth of the IPL (2–4). Basically, 5 different sub-layers of approximately equal thickness can be distinguished histologically (26) and with visible light OCT (27). Thus, it can be expected that this stratification of the IPL enables a differentiation of functional cell types without resolving individual cells by investigating the phase changes in different depths or sub-layers of the IPL.

## 2. Results

**Figure 1:**
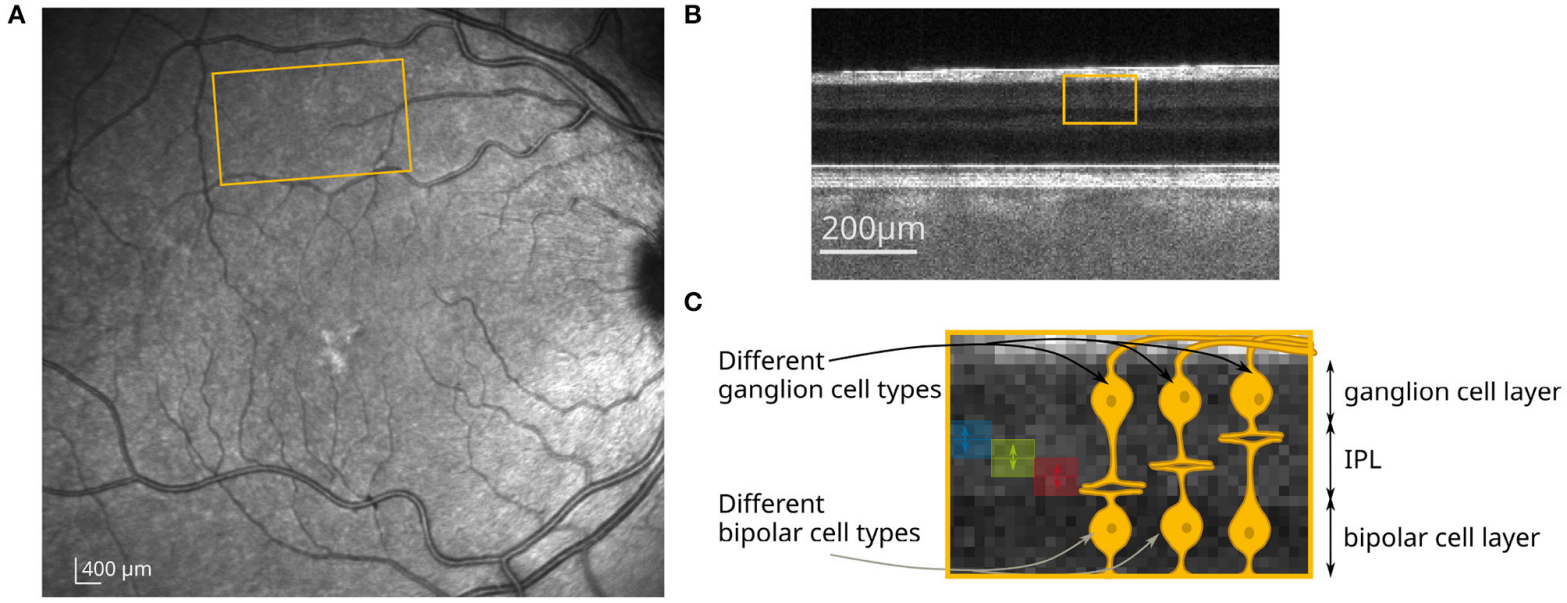
**(A)** Scanning laser ophthalmoscope (SLO) image at 815 nm (Spectralis, Heidelberg Engineering); yellow marked region corresponds to the field of view at 10° superior to the fovea. **(B)** B-scan of the retina imaged with an FF-SS-OCT system at this position. **(C)** Yellow marked region from **(B)** and close-up with a schematic drawing of the neuronal connections in the IPL. The different sub-layers of the IPL, for which the optical path length changes were obtained, are marked by the blue, green, and red areas.

**Figure 2:**
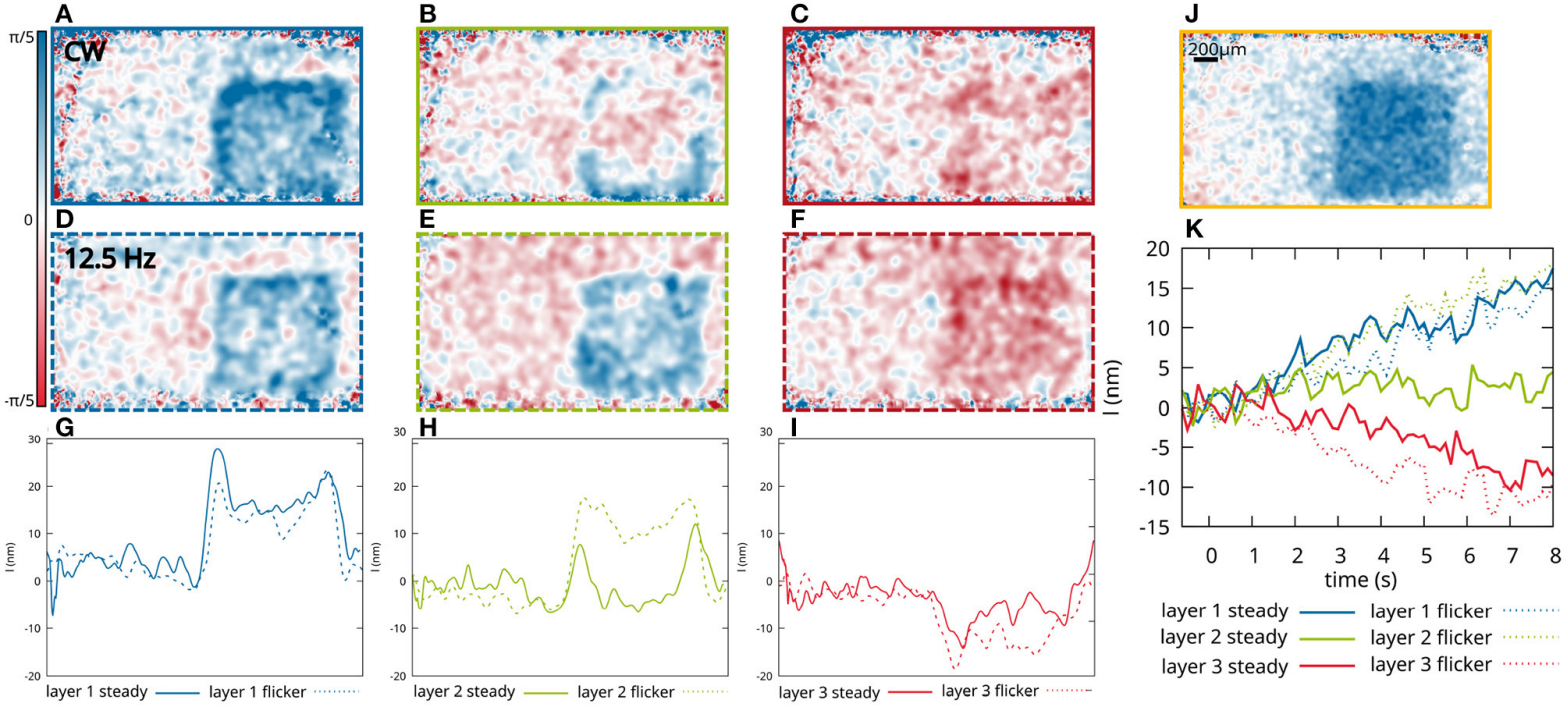
Functional changes of three different layers of the IPL as shown in Figure 1 after 8 s of stimulation with a square-shaped pattern. En-face images of the phase changes after 8 s of continuous stimulation, of the first layer **(A)**, the second layer **(B)** and the third layer **(C)**. En-face images of the optical path length changes after 8 s stimulation with a 12.5 Hz flickering light, of the first layer **(D)**, the second layer **(E)**, and the third layer **(F)**. **(G–I)** Profile of the optical path length change after 8 s stimulation. The phase changes are vertically averaged over 150 px (600 μm). **(J)** For comparison, phase changes in the photoreceptor OS are shown 0.5s after the beginning of the stimulation. **(K)** Corresponding time courses of the optical path length changes averaged over the central part of the stimulated area.

Here, the functional response of sub-layers to different visual stimuli was investigated in order to see if we can distinguish the response of different bipolar and ganglion cell types. During OCT imaging, the retina of a volunteer with no known diseases or ametropia was stimulated with a white LED combined with a transmission mask that projects a square-shaped pattern at 10° superior of the fovea on the retina (Figure 1A). At this position, very little distortion of the actual stimulation pattern and the functional OCT signal was observed (23) due to the wiring of the photoreceptors by the Henle fibers to the inner layers of the retina (28). In addition, at this position in the retina, the size of the dendritic fields of the ganglion cells are in the range between 100 and 200 μm for non-midget cells, or even lower for the midget cells (29). The size of the square shaped stimulation pattern is 1mm × 1mm, so that the resolution of the stimulated area in the IPL is expected to be sufficient to analyze the stimulation pattern. The irradiance on the retina was about 57nW/mm^2^, which corresponds to a luminance of 140cd/m^2^ and is therefore in the photopic vision regime (30). In order to investigate the functional phase change in different sub-layers of the IPL, the lower edge of the IPL was segmented (see Section 5 for detailed information). Phase differences were calculated in depths with constant distance to the segmented lower edge of the IPL. Due to the limited axial resolution of the OCT system, it was only possible to evaluate the optical path length change of three different sub-layers in the IPL, in which the 5 histologically differentiable layers partially overlap (see Figures 1B,C). The functional phase changes in the three different sub-layers after 8 s of continuous stimulation are shown in Figures 2A–C. For comparison, an en-face image showing the functional change in the photoreceptor OS of the same measurement is shown in Figure 2J. The response of the photoreceptors OS matches the essentially uniform stimulation pattern. The slightly washed out edges probably result from aberrations of the projection of the stimulation pattern on the retina and ocular motion. In contrast, the functional imaging of the IPL shows a pronounced signal at the edge of the stimulation pattern, while the optical path length changes in the center of the stimulation pattern are weaker for all three layers (Figures 2G–I). By this inhomogeneous behavior, the contrast at the edge regions of the stimulation pattern is increased and a prominent contour becomes visible. Furthermore, the functional changes in the sub-layers of the IPL also differ. The greatest change in the optical path length is in the inner sub-layer (closest to the ganglion cell layer). Here, a change in the optical path length of approx. 25nm is observed in the edges and an increase of approx. 15nm in the center. In the central layer, only a change in the edges of the stimulation pattern is observed (approx. 10nm), while no change is observed in the center. Additionally, in the outer layer, the optical path length decreases during stimulation. Here, it is not possible to distinguish between edges and center of the stimulation pattern. The time courses of the three layers are shown in Figure 2K (solid lines). The curves were obtained by averaging the phase differences over the central area of the stimulation pattern. Residual motion artifacts, caused for example by vascular pulsation, were corrected by subtracting a background that was obtained by averaging the phase change in an area in a safe distance (at least 200 μm) from the stimulation site.

In Figures 2D–F en-face images of the phase change for the three different layers after 8 s of flicker stimulation (square waveform of 12.5 Hz) with the same square pattern are shown. The corresponding time courses are shown in Figure 2K (dashed lines). Here, the functional changes in the inner sub-layer (blue, closest to the ganglion cells) and the outer layer (red, closest to the bipolar cells), do not differ significantly from the functional changes caused by continuous stimulation. However, a clear difference can be observed in the central layer. Here, the functional change under flicker stimulation is significantly increased, showing not only a stronger response in the edges of the stimulation pattern, but also a change in the center of the stimulation pattern, which shows an increase in the optical path length of about 15nm and is therefore about the same strength as the signal from the inner sub-layer.

**Figure 3:**
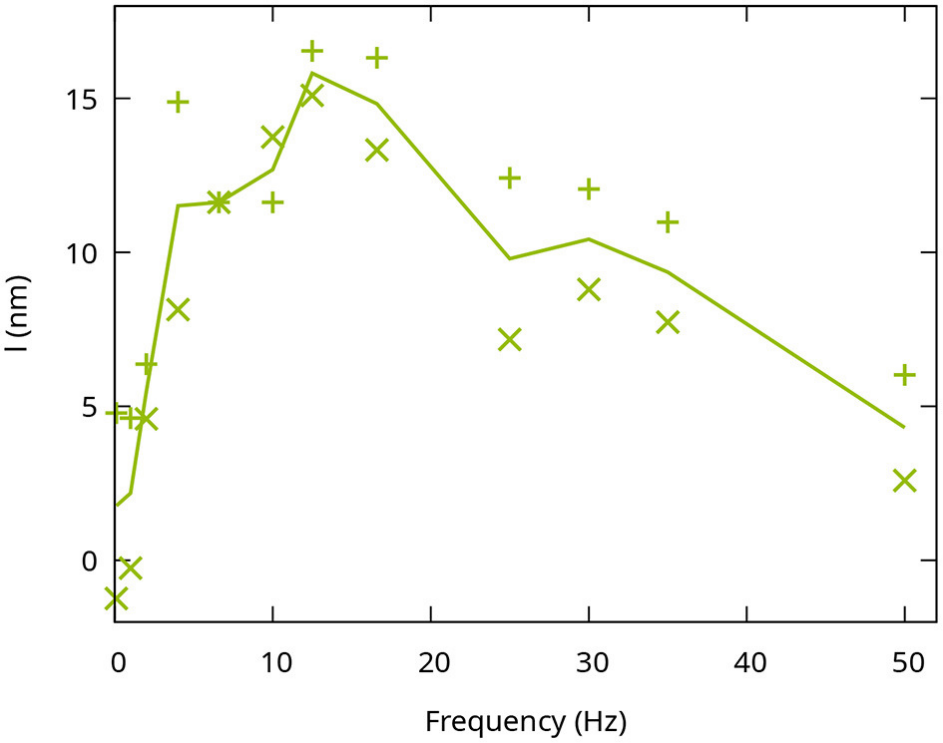
Frequency dependence of the functional phase change in the IPL. The optical path length changes I reached after 8 s were evaluated. Each point corresponds to an individual measurement. The line connects the average of the two measurements at each frequency.

Since the central layer is the only one that showed an increased response to flicker stimulation, it was investigated more closely to evaluate the sensitivity to different stimulation frequencies. In order to do so, the stimulation was performed with a continuous illumination and 11 different flicker frequencies of square waveform ranging from 1 to 55Hz with a duty cycle of 50%. Consequently, the number of photons reaching the retina was kept constant for all frequencies and halved compared to the constant stimulus. In order to quantify the sensitivity of the phase changes in the central IPL to the temporal frequencies, only the phase changes after 8s in the central part of the stimulation pattern were evaluated. Signal changes in the edge regions of the stimulation pattern were neglected for this investigation, since they were not effected by different stimulation frequencies in our observations. To reduce the influence of noise or remaining artifacts caused by the arterial pulsation, the optical path length changes over the last 625ms were averaged. Figure 3 shows the response of the central layer of the IPL to the different stimulation frequencies. As expected, continuous or low-frequency stimulation with 1Hz only resulted in a minimal change in the optical path length. The signal increased for higher frequencies (2−6.7Hz) and reached a maximum of around 15nm for stimulation frequencies of 12.5−16.6Hz. At even higher frequencies, the response decreased again and reached an optical path length change comparable to a continuous stimulation at 50Hz.

## 3. Discussion

For a functional evaluation of the phase in the human IPL *in vivo*, it is particularly important to have sufficient stability of the phase. Besides the general requirements for a phase-stable OCT system, as it is needed for the functional evaluation of the photoreceptors, a robust phase evaluation is crucial for a stable measurement of functional changes in the IPL, since a fast decorrelation of the speckle pattern is observed there, which is likely caused by metabolic processes in the inner retinal layers.

In contrast to other techniques that attempt to more directly assess retinal function and have been mostly used *ex vivo* or in animals, FF-SS-OCT relies on the changes in the phase of the backscattered light. The technique relies heavily on suitable post-processing of the data. As such, even on a state-of-the-art GPU (Radeon VII) accompanied by an Intel i7 8700 and 64 GB of RAM, the processing of the camera's data to suitably segmented OCT volumes takes almost 7 min, when excluding disc I/O. Afterwards almost 2 min are required for the phase evaluation (25), which is done on the CPU (Intel i7) and requires sufficient RAM to achieve this processing time.

The proposed technique is robust enough to be applied to the living human retina as it was demonstrated here. Previous techniques that classified and measured the different ganglion cell types are invasive and can only be applied in animal models or are even limited to cell cultures. An important disadvantages is still that it does not allow functional imaging of individual cells but rather has to rely on the stratification of the dendrite connections in the IPL.

The most important parts to obtain the results presented here is the phase-stable and fast data acquisition, combined with the phase evaluation taking statistical properties into account. In contrast, the exact algorithms used for registration and segmentation could be replaced by other algorithms, however, given the low SNR of the used OCT volumes, many existing solutions fail.

The observed functional changes can be well-explained by the stratification behavior of bipolar and ganglion cell types in the IPL. The OFF-bipolar cells, for instance, only connect in the outer layers (closest to the bipolar cells) with the OFF-ganglion cells, while the synaptic connection of the ON-bipolar and ganglion cells form in the inner layers (closest to the ganglion cells) (2, 3). Since the ON-cells get activated by light onset and the OFF-cells get inactivated, those two cell types react in opposite ways to the same visual stimulus. The increase of the optical path length observed here correlates with the stratification of the ON-bipolar and ganglion cells, while the observed decrease in optical path length coincides with the connection of OFF-bipolar and ganglion cells. Furthermore, we observed a high sensitivity to flickering light in the central layer of the IPL, whereas the response of the innermost and outermost layers was not affected by changes in time of the stimulation. This behavior fits well to the known stratification of sustained and transient cells. Sustained bipolar cells, which process slow visual changes, stratify on the inner and outer edges of the IPL, while transient bipolar cells, which process fast visual changes, tend to stratify in the middle of the IPL (2). Since the functional response in the inner and outermost layer does not change under flicker stimulation, those signals are probably caused by cell types that are sensitive to a different property in the visual signal such as intensity or chromatic information.

The temporal dependence of the IPL response of the middle layer with a maximum between 10 and 20 Hz and a return to the background above 50 Hz fits well to whole retina measurements using flicker ERG, electrical *ex-vivo* measurements on individual cells (31, 32), and measurements of increased blood flow (10). In all these experiments, a decrease in the functional contrast was observed for temporal frequencies at about 30 Hz and above. The frequency dependence measured here also follows the temporal contrast sensitivity curve of the human eye (33). It should be noted, however, that only slow changes in optical path length are measured here and that potential high-frequency changes in optical path length cannot be detected with the used volume rate (8 Hz). Furthermore, based on results obtained for photoreceptors by Tomczweski et al. (22), we expect the amplitude of the fast response to be more than one order of magnitude lower and, at these frequencies, it will be difficult to differentiate from heart-beat induced pulsation (34). Finally, while the observed response is linked to the neuronal activity, the time course of the observed path length changes may differ and show a slower temporal response.

A further interesting effect is the edge sensitivity of the functional changes across the stimulated area, which can be explained by the center-surround processing within the receptive fields of some bipolar and ganglion cell types (35, 36). In this processing scheme, stimulation of the marginal (surround) region of the receptive field acts antagonistically to stimulation in the central region. Areas in which the center of the receptive field and only part of the surrounding are stimulated thus produce a greater functional change than areas in which both the entire center and surround are stimulated. Such a processing scheme makes the cells particularly sensitive to edges in the visual signal (37). However, either the center activation has a higher influence than the surround inhibition, or additional cells contribute that lack the center-surround processing within their receptive field, causing a functional signal in the center of the stimulation pattern.

## 4. Conclusion

Here, we demonstrated that FF-SS-OCT combined with suitable post-processing provides sufficient phase stability and spatial resolution to analyze the neuronal signal processing in the human retina. Although, the functional changes of the optical path length in the IPL detected in this study can so far not be directly correlated with a specific physiological origin or molecular process, they are in good agreement with the existing knowledge of the visual signal processing of bipolar and ganglion cells.

It seems promising that phase-sensitive FF-SS-OCT can be used in the future to study the function of different, so far unclassified cell types *in vivo*. This could help monitoring innovative clinical treatments for ophthalmic diseases such as stem cell or optogenetic therapy in the retina (38, 39). Given the currently time-consuming nature of the measurements presented here, future studies will be required to simplify the procedure, confirm robustness and reproducibility of the signals in multiple individuals, and to evaluate the physiological parameters that can be extracted.

Differentiating functional groups of ganglion and bipolar cells does not need to resolve individual cells, but is possible by studying signals from different depths in the IPL, where their synaptic connections are stratified. This work thus demonstrates the potential of phase-sensitive OCT to study complex neurological function and signal processing *in vivo*, which opens avenues for functional studies in humans.

## 5. Methods

### 5.1. Setup

All measurement were done with a Michelson-interferometer based FF-SS-OCT [see e.g., (40–42)]. It uses a tunable light source (Superlum Broadsweeper, BS-840-1) with 51nm tuning range centered at λ_0_ = 841nm, resulting in an axial resolution of 8.4 μm (FWHM) in air. The light source illuminates an area of 2.6mm × 1.5mm on the retina. The total radiant flux on the retina from the sample illumination was about 5mW. The light backscattered by the retina was superimposed with a collimated reference beam and detected by an ultra-high speed camera (Fastcam SA-Z, Photron) at a frame rate of 60kHz at 640 × 368pixels. During the wavelength scan the camera acquired 512 images per volume, which results in a volume rate of 117 Hz at full duty cycle. The data had to be stored with 8-bit precision on an internal storage of about 8 GB. With the image size used and the number of frames required for each volume, this ultimately restricts the number of volumes in one imaging sequence to about 70. To achieve 8s of measurement time, a volume was triggered only every 125ms, corresponding to a volume rate of 8 Hz. Longer time intervals were hardly useful, because macroscopic and microscopic motion frequently destroyed the phase correlation between successive volumes.

During measurements, the retina was stimulated with a white LED and a transmission mask, which projected a square pattern on the retina. The stimulation light was coupled *via* a cold mirror into the sample arm and an infrared long pass filter in front of the camera ensured that no stimulation light disturbed the OCT imaging. The stimulation irradiance was about 57 nW/mm. The conversion from the irradiance $E_{e}\left[\frac{\text{W}}{{\text{m}}^{2}}\right]$, which was obtained from the spectrum of the used LED, to the illuminance $E_{v}\left[\frac{\text{lm}}{{\text{m}}^{2}}\right]$ is done by the following equation:


(1) $$\begin{array}{l} E_{v}=683\frac{\text{lm}}{\text{W}}\overset{\infty }{\underset{0}{\int }}\text{d}\lambda V\left(\lambda \right)E_{e}\left(\lambda \right) \end{array}$$

Here, *V*(λ) is the luminous efficiency function (43) and $683\frac{\text{lm}}{\text{W}}$ is a conversion factor. The conversion to luminance $L_{v}\left[\frac{\text{cd}}{m^{2}}\right]$ is done according to


(2) $$\begin{array}{l} L_{v}=E_{v}\frac{D^{2}}{A_{p}}\text{,} \end{array}$$

where *A*_*p*_ is the pupil area for a dilated pupil (50mm^2^) and *D* = 17mm is the distance from the pupil to the retina.

### 5.2. Measurements

All measurements shown here were carried out on a single female volunteer with no known diseases or ametropia. Written informed consent was obtained. Compliance with the maximum permissible exposure (MPE) of the retina and all relevant safety rules was confirmed by the ethics board of the University of Lübeck. All methods and measurements were performed in accordance with the relevant guidelines and regulations. The study was approved by the ethics board of the University of Lübeck (ethics approval Ethik-Kommission Lübeck 16-080). To optimize the light detection, the pupil of the volunteer was dilated by mydriatic eye drops to a diameter of approximately 8mm. Before the beginning of each measurement session, the subject was dark-adapted for at least 20 min and all measurements were performed in a darkened room. Between measurements, a break of at least 5 min enabled a complete regeneration of the stimulated area in the retina. All stimulation (continuous and flicker) started at the 5th recorded volume, and continued for the full duration of the measurement, so that the first 4 volumes served as baseline.

### 5.3. Data Processing

After the reconstruction of the OCT data, numerical corrections for axial motion, dispersion (44), and aberrations (41, 45) were done for all 70 volumes in a dataset.

#### 5.3.1. Registration

Afterwards, the movement between successive volumes was compensated by a non-rigid registration of the data. The registration utilizes correlation of sub-areas of the different volumes to determine their relative displacement and finally interpolates a three-dimensional displacement map between all volumes.

#### 5.3.2. Segmentation

In order to investigate the functional changes in different layers of the IPL, the lower edge of the IPL was segmented in a volume after aligning the volumes according to their registration. The utilized segmentation was loosely based on the work of Kafieh et al. (46). First, data points with a particularly high axial gradient were selected from a coarse-grained volume. Afterwards, a 3D-graph based algorithm called diffusion maps (47) was used. This algorithm maps the selected data points into a new coordinate system, where they are rearranged according their connectivity, i.e., based on their transition probability after multiple time-steps given only their transition probability in a single time-step. The single time-step transition probability of the graph was thereby determined from the local proximity of the data points to each other and the local curvature of the surface, assuming that transitions along a surface curvature are more likely than those that deviate from the local curvature. The mapped data points are then clustered by a density-based algorithm, called DBSCAN (48), separating them into different layers and finally interpolated to give the respective layers in the high resolution dataset.

Based on the segmentation, the curvature of all 70 complex-valued volumes was removed using the Fourier shift theorem and the individual A-scans were aligned, resulting in the lower edge of the IPL being flat.

#### 5.3.3. Phase Evaluation

After segmenting, the phase changes of the volumes were evaluated for different sub-layers. This phase evaluation was done by using an adaption of the extended Knox-Thompson algorithm (49–52), which is described in more detail elsewhere (25). For the evaluation, the cross-spectrum Δ*U*(*x, y, z, t*, Δ*t*) is calculated, which contains all possible temporal phase differences between the times *t* and *t*+Δ*t* for each position (*x, y, z*),


(3) $$\begin{array}{l} \Delta U\left(x,y,z;t,\Delta t\right)=U\left(x,y,z,t\right)U^{*}\left(x,y,z,t+\Delta t\right), \end{array}$$

where *U*(*x, y, z, t*) represents the complex-valued OCT volumes.

Following, the spatial phase-differences were calculated between two layers *Z*_1_ and *Z*_2_, each consisting of two voxels in depth, with constant distance to the segmented lower IPL edge. In order to receive a better SNR the phase-information for each layer were averaged over the 2 voxels in depth, according to


(4) $$\begin{array}{l} \Delta U^{Z_{1}-Z_{2}}(x,y;t,\Delta t)=\left(\sum_{z\in Z_{1}}\Delta U(x,y,z;t,\Delta t)\right)\\ {\left(\sum_{z\in Z_{2}}\Delta U(x,y,z;t,\Delta t)\right)}^{*} \end{array}$$

In the upper layers of the retina, the phase evaluation is especially challenging, since metabolic processes and blood flow cause strong molecular movements here. For this reason, decorrelation of the speckles occurs very fast and makes the phase evaluation difficult.

To minimize this kind of noise, an ensemble average of the phase differences over several adjacent lateral points is formed by convolving the phase with a lateral Gaussian function according to


(5) $$\langle \Delta U^{Z_{1}-Z_{2}}(x,y;t,\Delta t)\rangle \approx G_{\sigma^{2}}(x,y)*\Delta U^{Z_{1}-Z_{2}}(x,y;t,\Delta t).$$

Here, * denotes a circular convolution and $G_{\sigma^{2}}\left(x,y\right)$ is a Gaussian function with standard deviation of σ = 4 pixels. The resulting, averaged cross-spectrum $\langle \Delta U^{Z_{1}-Z_{2}}\left(x,y;t,\Delta t\right)\rangle$ was then used to find a phase function ϕ(*x, y, t*) that is consistent with all entries of the cross-spectrum (25).

**Figure 4:**
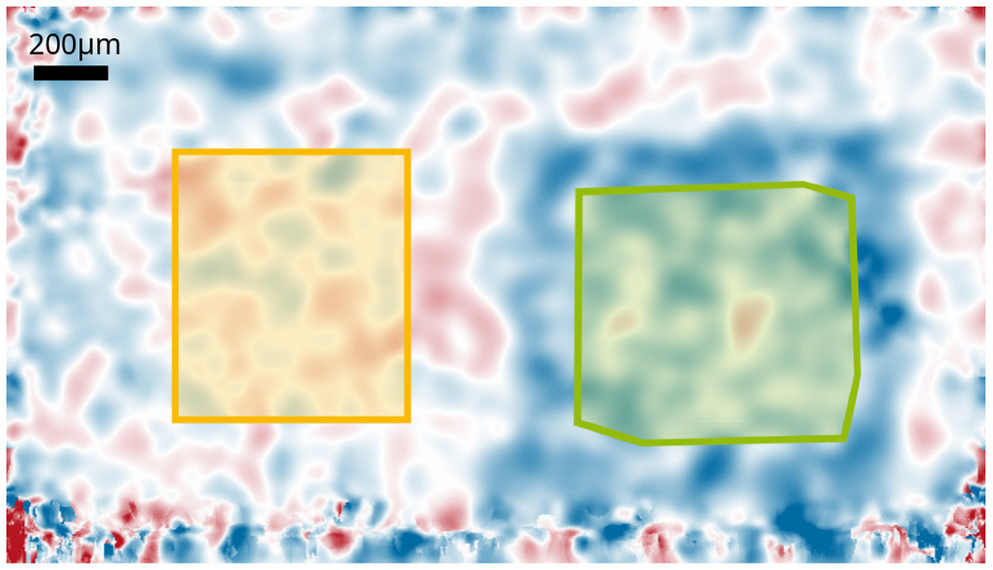
Phase changes of the central layer of the IPL after 8s stimulation with 12.5Hz. Areas used for the background corrections are indicated. The green marked area is the mask for the functional changes that is used to extract the time courses; the yellow marked area corresponds to the mask used to obtain the background noise.

From the resulting en-face phase images, the area in which a functional change occurs were determined. The phases in the area were averaged using an adapted mask to obtain a more stable ensemble average. Additional movement in the IPL, for example by vascular pulsation and other metabolic processes resulted in a continuously changing and non-reproducible background, which is superimposed to the functional signal. For correction and removal of the background noise, the phase changes in a second area that was not stimulated was subtracted from the functional changes (see Figure 4). The mask for the functional change was obtained manually for each measurement since the stimulated area could change slightly. The mask for the background noise was the same for all measurements.

## Data Availability Statement

The raw data supporting the conclusions of this article will be made available by the authors, without undue reservation.

## Ethics Statement

The studies involving human participants were reviewed and approved by Ethics board of the University of Lübeck. The participant provided written informed consent to participate in this study.

## Author Contributions

CP worked on the setup, collected, analyzed, and interpreted the data, worked on the post-processing of the data, and wrote the manuscript. HS worked on the setup, collected the data, and worked on the post-processing. KG worked on the post-processing. SH, LP, and DM worked on the setup and collected the data. YM interpreted the data. GH obtained the funding and interpreted the data. DH obtained funding, worked on the post-processing, and interpreted the data. All authors contributed to the article and approved the submitted version.

## Funding

This work was funded by the German Research Foundation (DFG), Project Number 629/6-1 and the Federal Ministry of Education and Research (BMBF 13N15432).

## Conflict of Interest

During the research LP was employed by the Medical Laser Center Lübeck and DH was an employee of Thorlabs GmbH. DH is also listed as inventor on a related patent application. GH is listed as inventor on a related patent application. The remaining authors declare that the research was conducted in the absence of any commercial or financial relationships that could be construed as a potential conflict of interest.

## Publisher's Note

All claims expressed in this article are solely those of the authors and do not necessarily represent those of their affiliated organizations, or those of the publisher, the editors and the reviewers. Any product that may be evaluated in this article, or claim that may be made by its manufacturer, is not guaranteed or endorsed by the publisher.
